# Novel program for automatic calculation of EPG variables

**DOI:** 10.1093/jisesa/ieae063

**Published:** 2024-06-28

**Authors:** Elisa Garzo, Antonio Jesús Álvarez, Aránzazu Moreno, Gregory P Walker, W Fred Tjallingii, Alberto Fereres

**Affiliations:** Departamento de Protección de Cultivos, Instituto de Ciencias Agrarias, CSIC, Madrid, Spain; Departamento de Ingeniería, Universidad de Almería, Almería, Spain; Departamento de Protección de Cultivos, Instituto de Ciencias Agrarias, CSIC, Madrid, Spain; Department of Entomology, University of California, Riverside, CA 92521, USA; EPG Systems, Wageningen, The Netherlands; Departamento de Protección de Cultivos, Instituto de Ciencias Agrarias, CSIC, Madrid, Spain

**Keywords:** electrical penetration graph technique, phloem feeder, workbook, feeding behavior, truncate wave criterion

## Abstract

The electrical penetration graph (EPG) technique is the most powerful tool for studying the feeding behavior of pierce-sucking insects. However, calculating EPG variables is often very time-consuming, and consequently, several software programs have been developed for the automatic calculation of EPG variables. Here we present a new user-friendly Excel Workbook that uses a standardized list of EPG variables and follows expert guidelines for calculating them. The program developed in Visual Basic for Applications (VBA) is a step up from the existing software and allows easy data analysis and interpretation. It also includes a novel option for dealing with the common problem of “truncated”—waveforms artificially terminated by the end of recording. The only requirement to run the program is Microsoft Excel software running under a PC environment. The Workbook was validated by calculating variables from EPG recordings of aphids and psyllids and the results obtained were compared with those of existing software such as the Sarria Workbook. Our EPG Workbook provides researchers with a reliable and standardized tool for the automatic calculation of up to 127 EPG variables from phloem-sap-sucking insects.

## Introduction

Hemipterans cause serious economic losses in a wide variety of crops, and many are vectors of plant pathogens (Perilla-Henao and Casteel 2016). The Order Hemiptera includes small sap-sucking insect vectors such as aphids, whiteflies, mealybugs, and psyllids. Although the probing and feeding behavior of piercing-sucking insects is not directly observable, these activities can be monitored indirectly by means of electronic devices.

McLean and Kinsey (1964) were the first to develop an “Electronic Monitoring System” (EMS) that could record the feeding behavior of aphids in real time using an alternating current (AC) circuit where the insect and the plant are part of an electrical circuit. The EMS revolutionized the study of piercing-sucking insects. Since its development, it has become the most accurate and direct method by which the probing behavior of piercing-sucking insects can be quantified and studied as it is occurring, thus, providing a clearer view of probing and feeding behavior than any other technique (Backus 1994). The EMS employs 2 electrodes, one connected to the plant, (usually in the soil of a potted plant), and the other connected to a pierce-sucking insect with an electrically conductive adhesive. Once the insect inserts its stylets into the plant, the circuit is closed and the electrical fluctuations across the insect–plant interface are recorded as characteristic waveforms representing different stylet penetrating activities through plant tissue. This first device was designed in California from 1962 to 1964 to study probing and feeding activities associated with the acquisition and inoculation of aphid-transmitted plant viruses (Backus 1994).

Years later, Tjallingii (1978, 1985) modified the original AC system by using a direct current (DC) circuit and renamed the technique as “electrical penetration graph” (EPG). Both monitoring systems (AC and DC) can measure resistance (*R*) changes in the plant–insect combination. In addition, the DC system also measures voltage changes originating in the insect and plant, called electromotive force (emf) fluctuations. In 2009, a new EPG AC–DC correlation monitor was designed to record either AC or DC signals with flexible input resistors to detect both *R* and emf components for piercing-sucking insects (Backus and Bennett 2009). This monitor provides double *R*-component measurements (AC and DC) that may interfere with each other, though mostly not disturbing waveform identification.

The EPG technique has mainly been used on hemipterans but since its origin, it has been extended to other insect orders—Thysanoptera (Kindt et al. 2006) and Diptera—(Wayadande et al. 2020), as well as some Acari (mites) (Li et al. 2019). The EPG technique has been extensively used to investigate insect–plant interactions, including the identification and characterization of host plant resistance to hemipterans (van Helden and Tjallingii 1993, Garzo et al. 2002, 2018, Alvarez et al. 2006, Sun et al. 2018, Escudero-Martinez et al. 2021, González et al. 2022), transmission mechanisms of plant pathogens by their insect vectors (Prado and Tjallingii 1994, Martin et al. 1997, Jimenez et al. 2018, 2020, Cornara et al. 2020), and the mode of action of pesticides including their antifeeding properties (Harrewijn and Kayser 1997, Costa et al. 2011, He et al. 2011, Butler et al. 2012, Garzo et al. 2020). This technique has also been used in combination with an artificial sieve-tube system to identify cues detected by aphids in sieve elements that trigger changes in feeding behavior (Will et al. 2008) and in combination with stylectomy to collect phloem sap (Gutiérrez et al. 2012, Garzo et al. 2023), as well as histological techniques to locate the stylets within the plant tissue (Tjallingii and Hogen Esch 1993, Garzo et al. 2018, Chinchilla-Ramírez et al. 2021).

Currently, EPG data acquisition (signal recording) uses specific free software for Windows platforms such as “Stylet+” software (EPG Systems, Wageningen, the Netherlands; www.epgsystems.eu/) or “Windaq Pro+” software (DATAQ Instruments Inc., Akron, OH). Identification and measurement of EPG waveforms in a recording followed by the calculation of EPG variables can be very time-consuming. Once the EPG data are processed, manual calculation of variables can be very tedious and complicated due to the large amount of EPG data in a recording. For example, aphids can perform more than 200 potential drops in an 8-h recording (Alvarez et al. 2013), and together with many other waveform periods, the amount of data coming from a single recording can be huge. Consequently, 7 computer programs have been developed for the automatic calculation of the most relevant EPG variables related to the probing and feeding behavior of piercing-sucking insects: (i) The “*EMIF workbook*” (Van Giessen and Jackson 1998); (ii) “*Sarria Workbook v5.0*” (Sarria et al. 2009); (iii) “*JKI*” (BAZ Excel Workbook for calculation of aphid EPG variables by Edgar Schliephake, unpublished); (iv) *EPG-Calc v6.1.7* (Giordanengo 2014); (v) “Backus v2.0” (unpublished); (vi) “*Ebert v2.0 SAS program for behavioral analysis of electrical penetration graph data*” (Ebert et al. 2015); and (vii) “*XylFeed v1.14*” specifically designed for xylem-feeders (Markheiser et al. 2022). However, these programs have limitations, such as discrepancies in their calculation methods, calculation errors, a limited number of files that can be processed at the same time, or the use of different acronyms for the same variable. Another limitation of these 7 computer programs is the way they handle the calculation of the mean and median duration of waveform periods when the final waveform is artificially truncated at the end of the recording. This can be a major problem because the real duration of the truncated waveform is unknown and consequently, the calculation of the mean and median waveform period duration is subject to error. The problem is most severe for waveforms that tend to occur only a few times during a recording and are also long-lasting, such as waveforms E1, E2, G, and F (Walker et al. 2024).

To address these limitations, the goal of our work was to develop a new EPG Workbook, referred to as the “*Novel Program for Automatic Calculation of EPG Variables (or NPAC-EPGv)*” to calculate the most relevant variables free of errors. Furthermore, the most significant improvement of this new EPG Workbook is that it includes the “truncated wave criteria” following the recommendations made by Walker et al. (2024) to calculate the mean and median values of waveform periods when the final waveform is artificially terminated by the end of the recording.

## Experimental Design

### Plants and Insects

EPG recordings of different hemipteran species were used to develop and then validate our New EPG Workbook (hereafter referred to as software “NPAC-EPGv”): (i) the potato aphid, *Macrosiphum euphorbiae* (Thomas) (Hemiptera: Aphididae), maintained on tomato plants (cv. Marmande) in a growth chamber at 23:18 °C (D:N); (ii) a colony of the African citrus psyllid, *Trioza erytreae* (Del Guercio) colony was maintained on lemon and bitter orange plant, as described by Benhadi-Marin et al. (2021).

### Electrical Monitoring of Insect Feeding Behaviosr

The probing and feeding behavior of *M. euporbiae* and *T. erytreae* were recorded using the DC-EPG system. We used 2 different devices, GIGA 4 and GIGA-8d for *M. euphorbiae* and *T. erytreae*, respectively (EPG Systems, the Netherlands). Young apterous adult aphids and adult female psyllids were immobilized by a vacuum device and a water-based silver conductive paint (EPG Systems, the Netherlands) was used to attach them to a thin gold wire (18.5 μm diameter), which was glued to a thicker copper wire (2 cm length). The EPG recordings were conducted on tomato plants for *M. euphorbiae* and on lemon plants for *T. erytreae* for 8 h using Stylet + software for Windows (EPG Systems, the Netherlands). To calculate the EPG variables, 5 EPG recordings were used for each insect species (aphid and psyllid) and processed using the new *NPAC-EPGv* software and the previously published Sarria Workbook software (Sarria et al. 2009) for comparison.

**Fig. 1.**
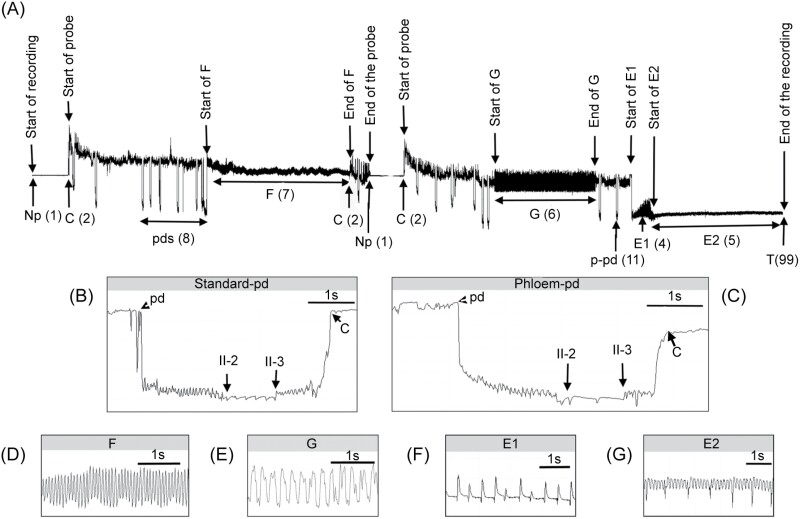
A) An 8-h EPG recording of *M. euphorbiae* feeding on tomato plants. Arrows below refer to the waveform labeling or marks (and their code numbers); arrows above indicate the start and end of probes and selected waveforms. B–G) Details of selected EPG waveforms (time scale shown in each subfigure): B) standard potential drop “waveform pd,” with subphases II-1, II-2, and II-3, C) phloem-potential drop “waveform p-pd,” the first contact with phloem tissue and its subphases, D) derailed stylet mechanics, “waveform F,” E) active intake of xylem sap, “waveform G,” F) salivation into phloem sieve elements, “waveform E1,” and G) passive phloem sap uptake from sieve elements, “waveform E2.”

The waveform interpretation was done according to previous EPG studies on the characterization of EPG waveforms of aphids (Kimmins and Tjallingii 1985, Tjallingii and Hogen Esch 1993, Prado and Tjallingii 1994, Chen et al. 1997, Jimenez et al. 2018, 2020) and psyllids (Bonani et al. 2009, Antolinez et al. 2017*).* The following waveforms were considered for analysis (Fig. 1):

▪ *Waveform np* represents non-probing behavior (no stylets contact with the leaf tissue).
▪ *Waveform C* represents the intercellular stylet pathway where the insects show a cyclic activity of mechanical stylet penetration and secretion of saliva.
▪ *Waveform potential drop (pd)* (included in waveform C) represents an intracellular stylet puncture. Two different pds were described: the standard pd and the phloem pd (p-pd) which is only reported in aphids and associated with stylet punctures in sieve elements and companion cells.
▪ *Waveform D* represents the first contact with the phloem. This waveform has only been reported for the Psyllidae family. This waveform is always preceded by C and usually finishes in an abrupt potential drop that marks the start of the salivation phase into a phloem sieve element (E1).
▪ *Waveform E1e* represents a putative extracellular watery salivation (Alvarez et al. 2006).
▪ *Waveform E1* represents salivation into phloem sieve elements at the beginning of the phloem phase and in between E2.
▪ *Waveform E2* represents passive phloem sap uptake from the sieve element (including salivation into the food canal, directly).
▪ *Waveform G* represents active intake of xylem sap.
▪ *Waveform F* represents derailed stylet mechanics.

In addition, for the validation of the “truncated waveform criteria,” we used 5 EPG data files that were artificially modified to include different numbers of E1-E2 periods (1, 2, or 3) and an artificially truncated E2 waveform in all recordings tested.

### NPAC-EPGv Software Development

The NPAC-EPGv software was developed under VBA environment for MS Excel and running on MS Windows. The software was structured into 10 user sheets (or screens) that serve as the user interfaces. These user sheets contain controls such as buttons, checkboxes, and option buttons, which generate waveform periods that trigger the execution of specific procedures. In addition, to the procedures written in the code editors of each sheet, there are 3 code modules responsible for importing and processing data, detecting errors in the data sequence, and performing calculations. These modules constitute the software’s structure.

## Procedures

### Select “Tools” Options and Open Data Files

**Fig. 2.**
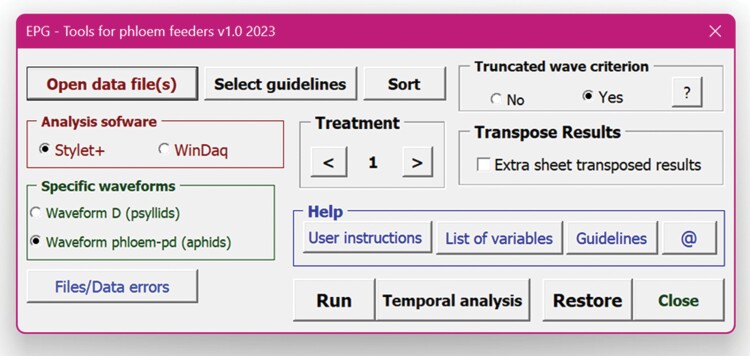
Tools panel of the NPAC-EPGv Workbook for automatic calculation of EPG variables.

**Fig. 3.**
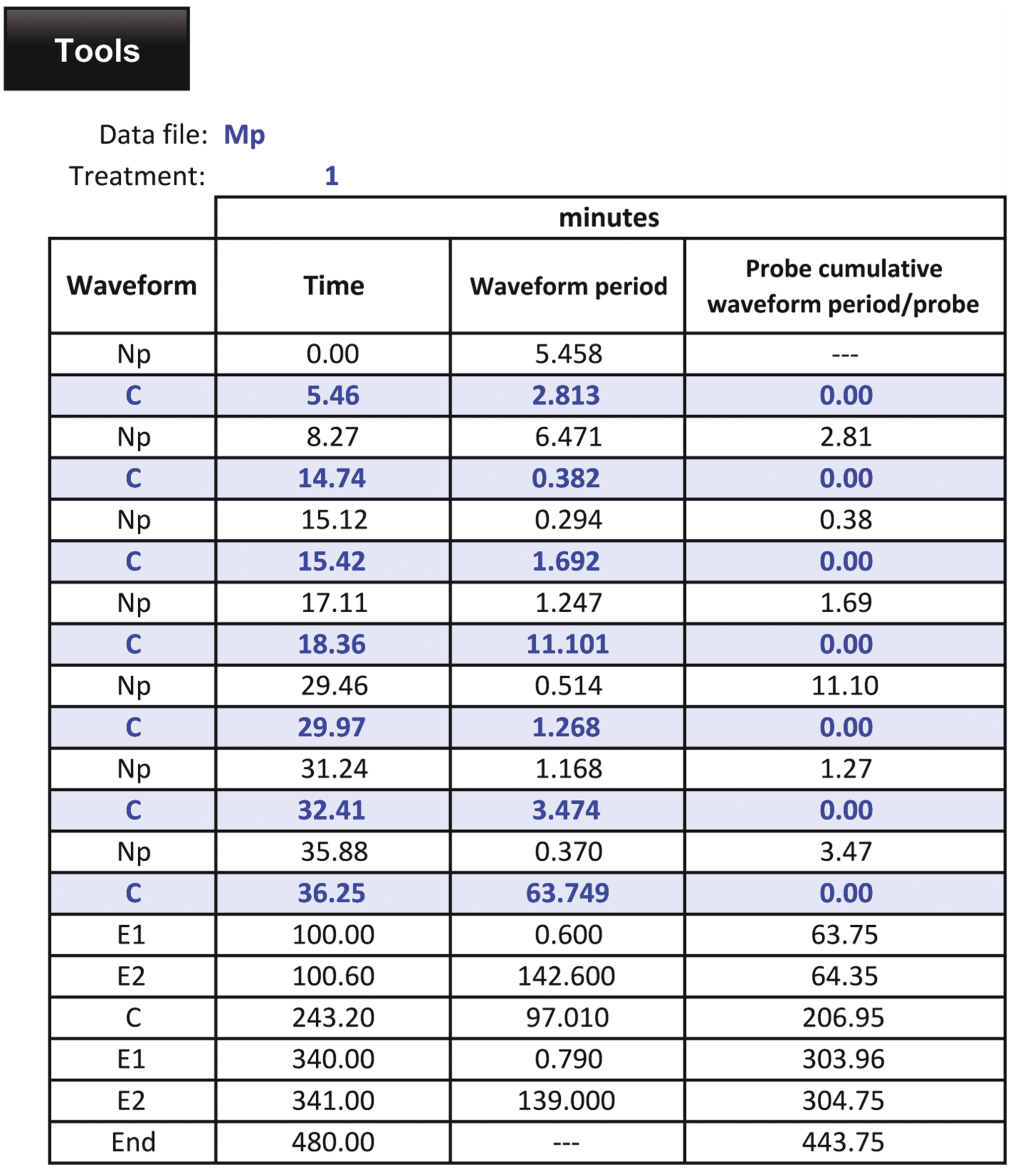
Data file displayed in the worksheet “Data.” Column 1 represents the sequence of waveforms. Each “Np” (non-probing) period represents the end of a probe, and rows that denote the start of a new probe (a row that starts with C) are highlighted; column 2 “*Time*” (in minutes from the start of the file) when each waveform begins; column 3, “*Waveform period*” is the duration of the waveform in the row. Column 4 “*Cumulative waveform period within a probe*” *is* calculated as the sum of the total waveform durations within a probe.

1.1. After opening the application, begin by clicking the [Tools] button, located at the top-left of the screen, which opens the “Tools panel” illustrated in Fig. 2.
1.2. The “Help” box, blue in the Tools panel (Fig. 2), gives access to the Instructions for using the application, the list of variables calculated by the application (see Supplementary Table S1), the Guidelines that must be followed to mark the waveforms in the recording (Table 1), and contact emails for support (Fig. 2 [Instructions], [List of variables], [Guidelines], and [@], respectively).
1.3. The first waveform marked on the recording must be np (non-probing). If the insect is already probing when the recording begins (e.g., EPG recordings with whitefly nymphs or mealybugs), users can click the [Select guidelines] button and then uncheck the first guideline “The recording is always starts with Np.”
1.4. The “Analysis software” box in the tools panel is used to select the EPG data acquisition software used for recording, either [Stylet+] or [Windaq Pro+].
1.5. In the “Specific waveforms” box select the waveform D for psyllids or phloem pd (p-pd) for aphids, if the user has previously marked either of these waveforms in the recording. Both waveforms (D or p-pd) use the same code number 11.
1.6. “Treatment” box is used to select the experimental treatments using numerical codes (1, 2, 3, etc.) and the EPG Data files are opened according to the treatment code. For example, select “1” in the “Treatment” box, click [Open data file(s)] button, and then open all the EPG data files for treatment 1. When dealing with a dataset for a different treatment, the number in the “Treatment” box must be changed accordingly.
1.7. After opening data files for all treatments, data are displayed in the “data” worksheet screen (Fig. 3). Each file displays the data file name and treatment number. The sequence of waveform periods is listed in column 1, and column 2 is the “*Time*” (in minutes from the start of the file) when a given waveform began. Column 3, “*Waveform period*”, is the duration of a given waveform in the row. Except for the first row, each “Np” (non-probing) represents the end of a probe, and rows that denote the start of a new probe (a row that follows an Np) are highlighted in blue. Column 4 represents the “*Cumulative waveform periods within a probe*” and is calculated as the sum of the total waveform durations within a probe.
1.8. By default, the calculated EPG variables will be displayed in the order seen in Supplementary Table S1. The user can change the display order before starting the calculation by clicking the [Sort] button, which will display the list of variables. The user can select variables from the list and move them up or down using the arrows. After arranging the variables in the desired order, users can save the file, and this display order of variables will be preserved for later use.
1.9. Clicking the [*Files/Data errors*] button will display the data file(s) that have errors with notes indicating the file number (position at which the file has been loaded), file name, and the cell (row number and column letter) where errors occur. The program is able to detect errors due to not following the guidelines described above or due to waveform duplication error (e.g., Np followed by E1 or Np followed by another Np sequence). The NPAC-EPGv will detect the error, and the message “You must review the sequence: ‘X’ wrong marks were detected” will appear. The cells with errors are highlighted in red.
1.10. The “*Truncated wave criterion*” box in the tools panel provides a recently developed protocol for calculating the median or mean duration of waveform periods when the last period of a waveform is truncated (artificially terminated) by the end of the recording (Walker et al. 2024). Refer to that paper for the details and justification of the protocol. A rough summary is provided here:

**Table 1.** Guidelines that the users must follow when marking the waveforms

#	Guidelines
1	The recording is always started with “Np”^a^
2	“Np” is always followed by “C”
3	“E2” is always preceded by “E1”
4	“pd waveform” is always preceded by “C” and followed by “C” or “Np”
5	The subphase sequence after pd (pdII-1) should be II-2, II-3
6	“F” is always preceded by “C”
7	“F” is always followed by “Np” or “C”
8	“G” is always preceded by “C”
9	“G” is always followed by “Np” or “C”
10	“E1” is always followed by “E1e,” “E2,” “C,” or “Np”
11	The end of each recording must be marked (T, code 99)

^a^If the insect is already probing when the recording begins (e.g., EPG recordings with whitefly nymphs or mealybugs), click the [Select guidelines] button in the “Tools panel” and then uncheck the first guideline “The recording is always started with Np.”

When the last period in the recording is truncated, the protocol estimates the “true median” period duration (the median if the true duration of the final period was known [i.e., not truncated]) based on where the duration of the truncated period ranks compared to the durations of the previous non-truncated periods. In some cases, an exact calculation of the true median can be made; for example, if there are 3 or more periods of the waveform and the truncated period is the longest, then the median calculated by including the truncated period will exactly be the true median. In this case, all that matters is that the truncated period is longer than the others; how much longer does not matter for calculating a median. In other cases, only the range of possible true median durations can be calculated. The truncated wave criterion option uses the middle of this range as a least biased estimate of the true median period duration. Depending on where the duration of the truncated period ranks in relation to the previous untruncated periods, a least biased estimate of the true median can be calculated either by including the truncated duration in estimating the median, excluding it, or by a formula based on the duration of the non-truncated periods. Details are given in Walker et al. (2024). When there are only 1 or 2 periods in the recording and the last period is truncated, calculating an accurate median duration is more problematic, but suggestions are given by Walker et al. (2024) and are followed when the truncated wave criterion is used.

Calculating mean period duration is more problematic than calculating median period duration when the last period is truncated because means are calculated solely on actual values. Unlike medians, the ranking of the truncated period relative to the previous non-truncated periods is irrelevant for calculating means. Walker et al. (2024) provide suggestions for calculating mean period duration when the last period is truncated, and those suggestions are followed when the truncated wave criterion is used.

The tools panel of the NPAC-EPGv software enables the user to select 1 of 2 options for dealing with truncated waveforms:

- If [No] is selected, then the truncated waveform is included in the calculation of the median (e.g., m_E2) and mean (e.g., a_E2) waveform period.
- If [Yes] is selected, the program will use an algorithm based on Walker et al. (2024) to estimate the median and mean period duration with minimal error when the last period of the waveform is truncated by the end of the recording.
1.11. Click the “Transpose Results” box, if users want an additional sheet “Results T,” where the results will be presented in transposed format.

### Running the Program and Calculation of EPG Variables

**Fig. 4.**
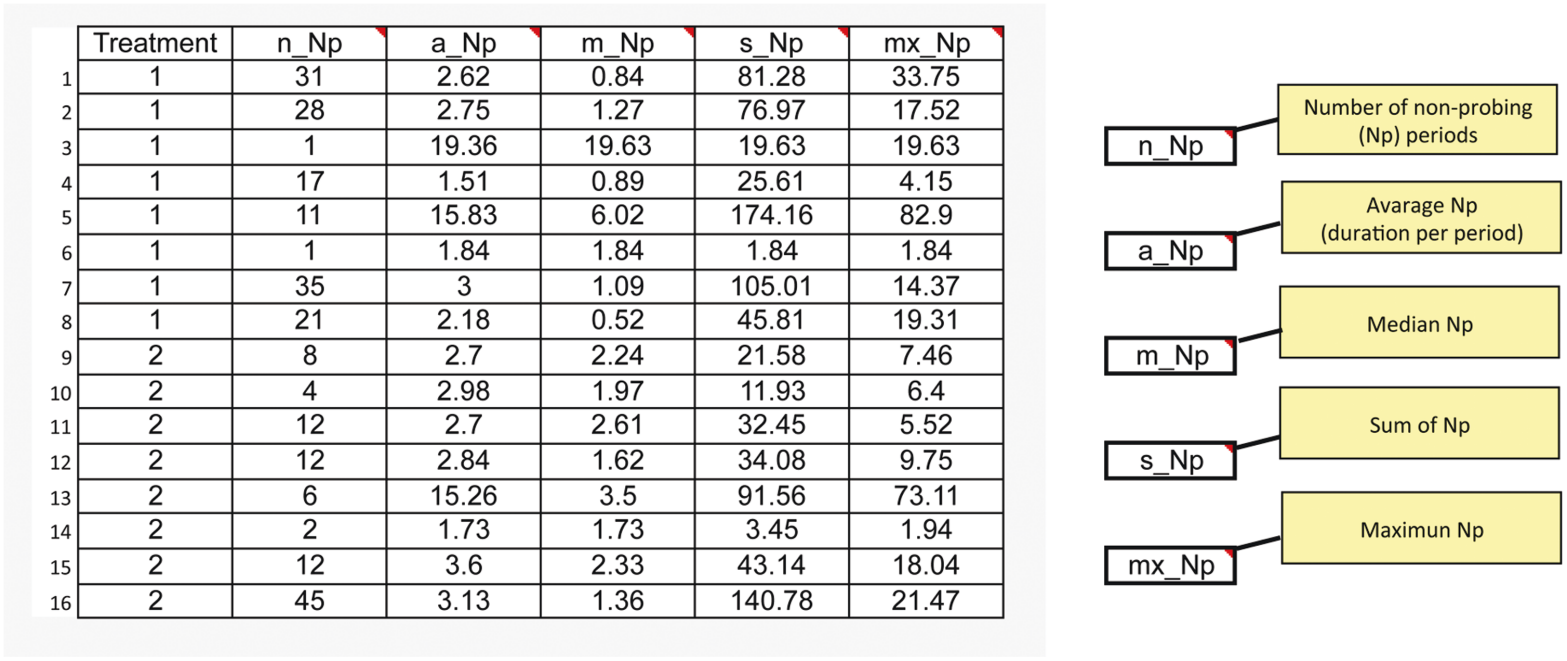
Details of the “Results” tab showing some of the EPG variables displayed. Each cell contains a comment (see boxes in the right of the figure) displaying the description of the variable.

2.1. Once the different options have been selected in the Tools panel then press the [Run] button to start the calculation of the EPG variables.
2.2. If there are no errors, the program displays the “Results” tab (Fig. 4) that displays the 127 calculated EPG variables. The variables names are given as abbreviations. To see a descriptive name, place the cursor on the cell with the abbreviated variable name, and a box with the full descriptive name will appear (Fig. 4).

The EPG variables are grouped by default as non-sequential and sequential variables (Van Helden and Tjallingii 1993; see Supplementary Tables S1 and S2):

- * Non-sequential variables* are those that do not consider the sequential order of the waveforms in the EPG recording. Non-sequential variables for each waveform include: *Number of waveform periods, mean, median, and total duration of the waveform.* These non-sequential variables are calculated for each individual recording. If a waveform of interest does not occur in a given recording, then its mean and median duration are considered as missing data.
- * Sequential variables* are measures of time or number of waveform periods that occur before or after a specified point in the recording. There are 4 general types of sequential variables: *“Time to a waveform period from a defined point”*; “*Number of waveform periods before or after a defined point”*; “*Duration of waveform periods before or after a defined point”; and “Potential indices.”*
a. “*Time to a waveform period from a defined point”* calculates time from a defined starting point to the start of a waveform. Commonly used starting points are (i) beginning of the recording (e.g., Time to first phloem phase (E1/E2) from the beginning of recording), if there are no phloem activities (E1/E2) these variables set to the total recording duration; (ii) beginning of first probe (e.g., Time to first phloem phase from the beginning of the first probe in the recording, if there are no phloem activities (E1/E2)) these variables set to the time from beginning of the 1st probe to end of recording; and (iii) beginning of the probe in which the waveform occurs (e.g., Time from the beginning of that probe to 1st E), if there are no phloem activities (E1/E2) these variables are a missing data.
b. *“Number of waveform periods before or after a defined point”* is another common type of sequential variable. For example, “Number of probes before 1st E in the recording” and “Number of brief probes (<3 min) before 1st E in the recording”; if there is no E, these variables set to the total number of probes or brief probes in the recording. For example, “Number of probes after 1st E” or “Number of probes after 1st sustained E2 (sE2) in the recording”. If there is no E or sE2 these variables set are missing data.
c. “*Duration of waveform periods before or after a defined point*” is a third type of sequential variable that calculates the total time, average time, or minimum time spent in a waveform before or after a defined waveform period. For example, “Time in C before 1stE in 1st probe with E” or “Minimum time in C to 1stE in probes with E.” If there is no E these variables set are missing data.
d. “*Potential indices*” were introduced by Montlor et al. (1983) and Girma et al. (1991). These indices measure the proportion of time that the insect engages in a waveform, but instead of being a proportion of the total recording time, these indices measure the duration of the waveform as a proportion of a subset of the total recording time. For example: time spent in waveform C as a proportion of total probing time. By eliminating non-probing time from the denominator, this index provides insight into the proportion of probing time occupied by waveform C rather than the proportion of total recording time. For example, “Potential E2 index” (proportion of time in E2 after the insect initiates its first E2) has been proposed as a variable for measuring phloem-based resistance. It is intended to measure phloem acceptability among treatments by comparing time spent ingesting phloem sap after eliminating pre-phloem resistance factors that would affect how long it takes to reach the first E2. However, it is still affected by pre-phloem resistance factors. If no E2 occurs, then this variable is set to missing data.

- Another results tab is called “Events,” where all waveform periods of a specific waveform per treatment are displayed in order to calculate the waveform duration per event (WDE), as described by Backus et al. (2007). However, multiple probes or waveform periods produced by the same insect are not independent, and treating them as such is pseudo-replication (Walker et al. 2024). The experimental unit (replicate) in EPG experiments is an individual EPG recording (one plant and one insect) (Van Helden and Tjallingii 2000). Thus, instead of WDE, the waveform duration per event (or waveform period) and per insect (WDEI) should be calculated for statistical comparisons between treatments.
- In addition, to gain insights into how behaviors change over time, the results can also be expressed either “by hour” or “cumulatively” by clicking the [Temporal analyses] button and selecting the hours that you want to evaluate. The “by-hour” option calculates variables separately for each hour of the recording (e.g., total time in E1 during the first hour, second hour, etc.). The “cumulative” option calculates variables from the start of recording to the end of the first hour, to the end of the second hour, etc.
- After the NPAC-EPGv software calculates the variables, users must save the output file using the “Save As” command on the File menu of MS Excel. After saving, they can click [Restore] button to clear all the data inserted into the workbook and analyze a new dataset.
- The output of the Excel worksheet results (values of the EPG variables of interest) for each particular insect and treatment can be exported, as Excel file, or copied and pasted to any statistical data analysis software (e.g., SPSS or R) for further analysis.

## Results

Both the NPAC-EPGv software and Sarria EPG Workbook (Sarria et al. 2009) were used to calculate EPG variables from the same aphid and psyllid EPG recordings. In total, 53 variables were calculated by both programs, and the results were identical. The variables common to both NPAC-EPGv and the Sarria Workbook are shown in Table 2. Manual calculation of the 74 variables unique to the NPAC-EPGv was performed using a hand calculator and gave the same results as the NPAC-EPGv. Thus, our new software provides reliable and automatic calculation of a large number of EPG variables.

**Table 2.** List of variables calculated in NPAC-EPGv and Sarria Workbook

Variable definition	Acronym	NPAC-EPGv	SARRIA
Number of periods of non-probing (Np)	n_Np	X	X
Average duration of Np periods	a_Np	X	X
Median duration of Np periods	m_Np	X	
Sum duration of all periods of Np	s_Np	X	X
Maximum Np period duration	mx_Np	X	
Duration of the second non-probe period	–	X	X
Total duration of non-probe before the 1st E	–	X	X
Duration of np just after the probe of the first sustained E2	–		X
Duration of np just after the probe of the first sustained E2 if that last non-probe reaches the end of the recording	–		X
Number of probes (Pr)	n_Pr	X	X
Average probe duration	a_Pr	X	
Median probe duration	m_Pr	X	
Sum duration of all probes	s_Pr	X	X
Duration of 1st probe	d_1Pr	X	X
Duration of 2nd probe	–		X
Number of short probes (< 3 min)	n_bPr	X	X
Number of periods of C	n_C	X	X
Average duration of C periods	a_C	X	X
Median duration of C periods	m_C	X	
Sum duration of all periods of C	s_C	X	X
Number of periods of F	n_F	X	X
Average duration of F periods	a_F	X	X
Median duration of F periods	m_F	X	
Sum duration of all periods of F	s_F	X	X
Number of periods of G	n_G	X	X
Average duration of G periods	a_G	X	X
Median duration G periods	m_G	X	
Sum duration of all periods of G	s_G	X	X
Number of periods of E1e	n_E1e	X	X
Average duration of E1e periods	a_E1e	X	X
Median duration of E1e periods	m_E1e	X	
Sum duration of all periods of E1e	s_E1e	X	X
Number of periods of D	n_D	X	
Average duration of D periods	a_D	X	
Median duration of D periods	M_D	X	
Sum duration of all periods of D	s_D	X	
Number of periods of single D	n_sgD	X	
Total duration of E (S_E12 + S_SGE)	–		X
Duration of the first phloem phase	d_1st_E	X	X
Number of periods of single E1	n_sgE1	X	X
Average duration sgE1 periods	a_sgE1	X	
Median duration sgE1 periods	m_sgE1	X	
Sum duration of all periods of sgE1	s_sgE1	X	X
Longest period of sgE1	mx_sgE1	X	
Number of periods of frE1	n_frE1	X	
Average duration of frE1 periods	a_frE1	X	
Median duration of frE1 periods	m_frE1	X	
Sum duration of all periods of frE1	s_frE1	X	
Longest period of frE1	mx_frE1	X	
Number of periods of E1	n_E1	X	X
Average duration of E1 periods	a_E1	X	X
Median duration of E1 periods	m_E1	X	
Sum duration of all periods of E1	s_E1	X	X
Longest period of E1	mx_E1	X	
Mean duration of initial E1 in phloem phase	a_1st E1_followed_E2	X	
Number of periods of E12	n_E12	X	
Average duration of E12 periods	a_E12	X	
Median duration of E12 periods	m_E12	X	
Sum duration of all periods of E12	s_E12	X	
Longest period of E12	mx_E12	X	
Number of periods of E2	n_E2	X	X
Average duration of E2 periods	a_E2	X	X
Median duration of E2 periods	m_E2	X	
Sum duration of all periods of E2	s_E2	X	X
Mean duration of E2 per phloem phase	a_s_E2/phloem_ph	X	
Longest period of E2	mx_E2	X	X
Duration of the 1st E2 in the recording	d_1st_E2	X	X
% insects with E2	%_E2/Tr	X	
Number of periods of sE2	n_sE2	X	X
Average duration of sE2 periods	a_sE2	X	
Median duration of sE2 periods	m_sE2	X	
Sum duration of all periods of sE2	s_sE2	X	
% insects with sE2	%_sE2/Tr	X	
Total duration of non-phloematic phase	–		X
Time to 1st probe from start of recording	t > 1Pr	X	X
Time to from start of EPG 1st sustained E2 (> 10 min)	–		X
Time from start of EPG to 1st E2	–		X
Time to 1st E from start of 1st probe	t > 1E	X	X
Time to the 1st E12 from start of 1st probe	t > 1E12	X	
Time to the 1st E2 from start of 1st probe	t > 1E2	X	X
Time to 1st sE2 from start of 1st probe	t > 1sE2	X	
Time from the beginning of that probe to 1st E	tPr > 1E/1Pr	X	X
Time from the beginning of that probe to 1st E2	tPr > 1E2/1Pr	X	X
Time from the beginning of that probe to 1st sE2	tPr > 1sE2/1Pr	X	X
Time in C to 1stE in 1st probe with E	tC > 1E/1Pr	X	
Time in C to 1st sE2 in 1st probe with sE2	tC > 1sE2/1Pr	X	
Average time in C to 1stE in probes with E	atC > 1E/Pr	X	
Minimum time in C to 1st E in probes with E	mntC > 1E/Pr	X	X
Total duration of non-probing before the 1st E in the recording	s_np.1E	X	X
Number of probes before 1st E in the recording	n_Pr > 1E	X	X
Number of brief probes (< 3min) before 1st E in the recording	n_brPr > 1E	X	X
Number of probes before 1st E2 in the recording	n_Pr > 1E2	X	
Number of probes before 1st sE2 in the recording	n_Pr > 1sE2	X	
Number of E2 before 1st sE2 in the recording	n_E2 > 1sE2	X	
Number of probes after 1st E in the recording	n_Pr.after1E	X	X
Number of brief probes (< 3 min) after 1st E in the recording	n_bPr.after1E	X	
Number of probes after 1st sE2 in the recording	n_ Pr < 1sE2	X	
Duration of E1 followed by E2	d_E1followedbyE2	X	
Duration of E1 followed by sE2	d_E1followedbysE2	X	
Duration of np just after the probe of the first sustained E2	–		X
Number of E1 (longer than 10 min) followed by E2	-		X
Duration of np just after the probe of the first sustained E2 if that last nonprobe reaches the end of the recording	–		X
Average duration of E1 followed by E2	–		X
Average duration of E1 followed by sustained E2	–		X
Duration of E1 followed by the first E2	–		X
Duration of E1 followed by first sustained E2 (> 10 min)	–		X
Contribution of E1 to phloem phase (%)	–		X
E2/C ratio	E2/C_ratio	X	
E1 index	E1_index	X	
E fractioning ratio	frE1_ratio	X	
E2 index	E2_index	X	X
% of probing time spent in C	%probtimeinC	X	X
% of probing time spent in F	%probtimeinF	X	X
% of probing time spent in G	%probtimeinG	X	X
% of probing time spent in E1	%probtimeinE1	X	X
% of probing time spent in E2	%probtimeinE2	X	X
% of E2s that are sustained E2s (> 10 min)	%_sE2	X	X
% of phloem phases that fail to achieve ingestion	%Phloem_ph_fail	X	
Number of pds	n_pd	X	X
Number of pds per minute of pathway phase	n_pd/minC	X	
Average duration of pds	a_pd	X	X
Median duration of pds	m_pd	X	
Sum duration of all pds	s_pd	X	X
Average pd II-1 duration	a_pd II-1	X	
Median pd II-1 duration	m_pd II-1	X	
Sum of time pd II-1 periods	s_pd II-1	X	
Average pd II-2 duration	a_pd II-2	X	
Median pd II-2 duration	m_pd II-2	X	
Sum of time pd II-2 periods	s_pd II-2	X	
Average pd II-3 duration	a_pd II-3	X	
Median pd II-3 duration	m_pd II-3	X	
Sum of time pd II-3 periods	s_pd II-3	X	
Time to 1st pd	t > 1pd	X	
Time to 1st pd in 1st probe	t > 1pd/1Pr	X	
Average time to 1st pd in a probe for all probes with pds	at > 1pd/Pr	X	
Median time to 1st pd in a probe for all probes with pds	m_Pr > 1pd	X	
Minimum time to 1st pd in a probe among all probes with pds	mnt_1pd/1pd	X	
Number of pds in 1st probe	n_pd/1Pr	X	
% probes with at least one pd	%_Pr_pd	X	
Number of probes before 1st pd	n_Pr > 1pd	X	
Sum duration of II-3 in 1st 5 pds	s_pdII-3/5pd	X	
Number of phloem pds	n_p-pd	X	
Average duration of p-pd	a_p-pd	X	
Median duration of p-pd	m_p-pd	X	
Total duration summed over all phloem pds	s_p-pd	X	
Time from the end of the last pd to the end of the probe	–		X
Time from the end of the last pd to the beginning of the E1 followed by the sustained E2 (> 10 min)	–		X
Time from the end of the last pd to the end of the EPG recording	–		X

## Discussion

It is important to highlight that the Sarria Workbook software v.5 (Sarria et al. 2009), on which our NPAC-EPGv software was based, is the most widely used workbook for the automatic calculation of EPG variables, with a total of 259 citations (Google scholar 2024a). Furthermore, *Sarria Workbook*, available at http://web.ua.es/masterbiodiversidad/descargas/EPG_analysisworksheet_v5.xls, is an Excel macro program runs under Visual Basic and can import data from various EPG data acquisition software: Probe (EPG Systems, Wageningen, the Netherlands) Stylet+, Windaq Pro+, and MacStylet v2.0 b10 (Febvay et al. 1996). The program is able to calculate up to 121 sequential and non-sequential variables and also identifies common errors in the data file’s waveform sequence, such as a waveform other than C following np, or an E2 that is not preceded by E1. In the present work, we describe our NPAC-EPGv, which imports data from Stylet+ and Windaq Pro+, which are the 2 most common programs currently used for EPG data acquisition (e.g., Roddee et al. 2023, Zippari et al. 2024). NPAC-EPGv calculates up to 127 EPG variables and identifies common errors that often occur when marking EPG data files. Our NPAC-EPGv does not provide a built-in statistical analysis and the output under the “Results” sheet needs to be exported to a statistical program for analysis.

Ebert et al. (2015) developed the “*Ebert v2.0*” program which is available at http://www.crec.ifas.ufl.edu/extension/epg/. This program is similar to the “Sarria Workbook” for aphids in terms of calculated variables and the order in which they are analyzed. The Ebert v2.0 program uses SAS to calculate the diverse suite of derived variables for aphids and provides statistical analysis via powerful mixed-model ANOVA using a single software platform. Ebert et al. (2015) mentioned that the Sarria program has the disadvantage of lacking a built-in statistical analysis (as does the NPAC-EPGv software). However, we do not see this as a disadvantage because not all researchers use the same statistical software (SAS, SPSS, R, etc.). The NPAC-EPGv software provides flexibility for the users to choose their preferred statistical software instead of being forced to use a specific statistical tool and method to manage and run statistical analysis of the EPG data output. The Ebert v.2.0 program is open code, so researchers can adapt this program to a particular study insect. However, the program is not user-friendly and requires users to install in its own computer and be familiar with SAS software to complete EPG data analysis. SAS is a very powerful statistical package but can be complicated to use and is expensive, thus, it might not be available to all users. Recently, many users have been using R statistical packages to run EPG data analysis as it is an open-access free software.

Another program commonly used for EPG analysis is *EPG-Calc v. 6.1.7*. This program was developed by Giordanengo in 2014 and cited by 62 users (Google scholar 2024b). It is a web-based application that offers a fast and user-friendly interface for calculating up to 100 EPG variables. In contrast, the NPAC-EPGv software can calculate 127 EPG variables of different treatments at the same time. The disadvantage of the *EPG-Calc* software is that the users must upload their data to the INRA website rather than running the software on their own computer.

The *JKI program* developed by Edgar Schliephake at the Julius Kühn-Institut, Germany, is an Excel Workbook that has similar properties to the programs described above. However, the *JKI program* has a useful feature: in addition to calculating the number of waveform periods, mean duration, and total duration of each EPG waveform for the entire recording, it also calculates these variables for each hour in the recording, revealing how these variables change over the course of the recording. These are referred to as “by hour” variables. The Sarria Workbook also calculates the number of waveform periods and duration of some waveforms in a by-hour basis. One advantage of our new NPAC-EPGv Workbook is that it also calculates the number of waveform periods, the mean and median waveform duration and total duration both cumulatively and by-hour ([Temporal analyses] button in “Tools” panel). An advantage of JKI and our NPAC-EPGv software is that the users can load all data files in one folder at the same time, whereas in the Sarria and EPG Cal programs, data files need to be loaded one by one.

Recently, a novel software designed as a new MS Excel macro called *XylFeed* v1.14 has been developed by Markheiser et al. (2022) to calculate DC-EPG waveform variables of xylem feeder insects such as spittlebugs and sharpshooters and can also calculate EPG variables in an hour-by-hour basis. The description of the software can be accessed at https://www.openagrar.de/receive/openagrar_mods_00083404, and can be freely downloaded. The above-mentioned software complements well our new NPAC-EPGv software, which was not designed specifically for spittlebugs and other xylem-feeders.

Another important addition is that our new NPAC-EPGv software includes the definition of standardized EPG variables and uses the same acronyms as described by W.F. Tjallingii in his standardized list (https://www.epgsystems.eu/downloads-install-files-manuals/file/38-list-of-parameters). Furthermore, information on the criteria used to calculate the EPG variables in our NPAC-EPGv software and some recommendations for handling common variables used in EPG studies, when the waveform of interest does not occur, have been added to the EPG variable list referred above. These additions can be found in the: “List of Standardized EPG Variables definitions & calculations” (Supplementary Table S1).

A novel feature of the new NPAC-EPGv program is that it offers an option for minimizing errors in estimating mean or median waveform period duration when the final waveform is artificially interrupted (truncated) by the end of the recording. When calculating the mean or median duration of a waveform, including a truncated waveform will always lead to an underestimate of the mean and often will underestimate the median as well. However, if the truncated waveform is very long despite being truncated, excluding it from calculating the mean or median waveform period duration often results in a greater underestimate than if it was included. In the “Tools” panel (Fig. 2), the user has the option of using this procedure (referred to as the “truncated wave criterion”). If the user does not use this option, then if there is a truncated waveform, it will be included in the calculation of mean and median duration.

The only requirement to run the NPAC-EPGv software is to have Microsoft Excel software installed on a PC. To run the application on a Mac OS computer, the user will must run it in a virtual Windows environment (e.g., Virtual Box).

Overall, our NPAC-EPGv Workbook provides researchers with a reliable and user-friendly tool for the automatic calculation of EPG variables that are commonly used in studies of the feeding behavior of sap-sucking insects.

A copy of this program is available at: https://epg.csic.es/downloads.

## Supplementary Material

ieae063_suppl_Supplementary_Table_S1

ieae063_suppl_Supplementary_Table_S2
