# Supplementary material for: Novel program for automatic calculation of EPG variables
Source: J Insect Sci. 2024 Jun 28;24(3):28. doi: 10.1093/jisesa/ieae063 (PMC11212364; doi:10.1093/jisesa/ieae063)
Supplement: ieae063_suppl_Supplementary_Table_S1 [file ieae063_suppl_supplementary_table_s1.docx]

**List of Standardized EPG Variables definitions & calculations**

**last update: 12/03/2024**

**Columns:**

**- #, number:** Variable number

**- Acronyms:** Abbreviated name of the variable

**- Variable definition:** description of the variable

**- Notes:** Further definition and/or explanation of the definition

**- Value when waveform does not occur:** How variables are calculated when the waveform(s) associated with the variable does not occur in the recording

**General remarks:**

Many variables are important indicators for plant geno- and phenotypic factors as well as for specific plant parts or tissues. Such factors may stimulate or inhibit waveform (aphid activity) occurrences, durations, or first time of appearance. Many variables are mutually dependent. It will often be better to present a selection of (the best) variables rather than all of them (listed here or elsewhere). Also, not only variables that differ are important but also some that are not different can provide important information.

**Artifacts affecting variable calculations:**

1. Average and median durations of waveform periods are affected when the last period is artificially cut short (truncated) by the end of recording. The truncated wave criterion discussed in the Instructions provides an option for minimizing error imposed by truncated waveform period when calculating median and mean duration of the waveform period.

2. In sequential variables, the time to the first occurrence of a certain waveform cannot be calculated in recordings (replicates) in which that waveform does not occur. For example, the time to the first E (phloem phase) in recordings without any E. There are 3 options for scoring the variable "time to first occurrence" of a waveform when the waveform does not occur: Option 1) Exclude replicates where the waveform does not occur (i.e., "time to first occurrence" = missing data ). However, in most cases, it is likely that the waveform would eventually have occurred if the recording period was longer; in other words, non-occurrence of a waveform is likely due to the recording period being too short. In that case, excluding replicates where the waveform did not occur, leaves only replicates where the insects were relatively quick to produce the first occurrence of the waveform, resulting in an underestimate of the average time to first occurrence of the waveform. Option 2) Enter "0" for time to first occurrence of the waveform; however, this would lead to an even more severe underestimate of time to first occurrence of the waveform. Option 3) If we assume that the non-occurrence of a waveform was due to the recording period being too short, then we know that the time to first occurrence of the waveform is at least as long as the recording period. With this assumption, the time to first occurrence of the waveform can be entered as the total recording time (or time from 1st probe until the end of recording). This will still result in an underestimate of mean time to first occurrence of the waveform, but will be less of an underestimate than options 1 and 2. Furthermore, if the number of replicates that lack the waveform is less than half the total number of replicates and option 3 is used, then *median* time to first occurrence of the waveform will be accurate and unbiased. The workbook uses option 3. Alternatively, "percentage of insects that produce the waveform" could be used as a substitute statistic that provides a similar biological perspective as "time to first occurrence of the waveform"

**Consideration of "total time in waveform" variables**

One of the non-sequential variables associated with each waveform is "Total duration" of the waveform summed over all periods of the waveform in the recording. Total durations of different waveforms are interdependent on each other: more time in one waveform leaves less available time in the recording for other waveforms. This needs to be taken into consideration when interpreting total time in a waveform. For example, a shorter total time engaged in phloem sap ingestion (waveform E2) by itself does not indicate lesser phloem sap quality or acceptability. Shorter total time in E2 also could be due to longer times in other waveforms such as pathway (waveform C) or xylem ingestion (waveform G). Median or mean duration of E2 period would be a better indicator of phloem sap quality or acceptability.

**NON-SEQUENTIAL VARIABLES**

Non-sequential variables include number of periods (occurrences) of a waveform in the recording, the sum duration of all the periods of the waveform, and average and median duration of waveform period. Distribution of period durations are often skewed toward longer durations, especially for waveform E2; consequently, means and medians sometimes may differ considerably. If statistical comparisons among treatments yield different results for mean and median duration of the waveform periods, then inspection of the distribution of period duration may provide insights that will facilitate more accurate biological conclusions.

| Number # | Acronym | | | Variable definition | | Notes | | Value when waveform does not occur |
| --- | --- | --- | --- | --- | --- | --- | --- | --- |
| **"Non-probing"** | | | | | | | | |
| 1 | n_Np | | | | number of periods of non-probing (Np) | includes the 1st and the last Np. | |  |
| 2 | a_Np | | | | average duration of Np periods |  | | If there are no Np periods, then  a_Np = missing data |
| 3 | m_Np | | | | median duration of Np periods |  | | If there are no Np periods, then  m_Np = missing data |
| 4 | s_Np | | | | sum duration of all periods of Np | If >75% of recording is Np, then discard replicate | |  |
| 5 | mx_Np | | | | maximum Np period duration |  | | If there are no Np periods, then  mx_Np = missing data |
| **"Probing"** | | | | | | | | |
| 6 | n_Pr | | | | number of probes (Pr) |  | |  |
| 7 | a_Pr | | | | average probe duration |  | |  |
| 8 | m_Pr | | | | median probe duration |  | |  |
| 9 | s_Pr | | | | sum duration of all probes | s_Pr + s_Np = total recording time | |  |
| 10 | d_1Pr | | | | duration of 1st probe |  | |  |
| 11 | n_bPr | | | | number of short probes (<3min) |  | |  |
| **"Pathway"** | | | | | | | | |
| 12 | n_C | | | | number of periods of C | C is considered as all waveforms A, B, C and includes all pds; it does NOT include E1e, F and G | |  |
| 13 | a_C | | | | average duration of C periods |  | |  |
| 14 | m_C | | | | median duration of C periods |  | |  |
| 15 | s_C | | | | sum duration of all periods of C |  | |  |
| **"Derailed stylet mechanics"** | | | | | | | | |
| 16 | n_F | | | | number of periods of F | sometimes G starts at a high frequency similar to F, but should not be scored as F | |  |
| 17 | a_F | | | | average duration of F periods |  | | If there are no F periods, then  a_F = missing data |
| 18 | m_F | | | | median duration of F periods |  | | If there are no F periods, then  m_F = missing data |
| 19 | s_F | | | | sum duration of all periods of F |  | |  |
| **"Xylem"** | | | | | | | | |
| 20 | n_G | | | | number of periods of G | G reflects water stress and mainly seems an indicator for thirst. | |  |
| 21 | a_G | | | | average duration of G periods |  | | If there are no G periods, then  a_G = missing data |
| 22 | m_G | | | | median duration G periods |  | | If there are no G periods, then  m_G = missing data |
| 23 | s_G | | | | sum duration of all periods of G |  | |  |
| **"Pathway salivation"??** | | | | | | | | |
| 24 | n_E1e | | | | number of periods of E1e |  | |  |
| 25 | a_E1e | | | | average duration of E1e periods |  | | If there are no E1e periods, then  a_E1e = missing data |
| 26 | m_E1e | | | | median duration of E1e periods |  | | If there are no E1e periods, then  m_E1e = missing data |
| 27 | s_E1e | | | | sum duration of all periods of E1e |  | |  |
| **"Phloem phase"**  Phloem phase (E) is comprised of waveform E1 (salivation into the sieve element) and E2 (ingestion of phloem sap). Phloem phase always starts with E1 and may or may not be followed by E2. E12 refers to phloem phases that have both E1 and E2. Waveform E1 also can occur later in phloem phase following E2 or interrupting E2. The term "E1 fractions" (frE) refers to all periods of E1 that occur within an E12 phloem phase. The variable sgE1 (single E1) refers to periods of E1 that are not followed by E2 (i.e., a phloem phase with only E1, no E2). E2 periods of > 10min are referred to as sustained E2 (sE2). | | | | | | | | |
| 28 | n_D | | | | number of periods of D | | Waveform D is specific to psyllids. Waveform D is always preceded by C and usually finishes in an abrupt potential drop that marks the start of E1. |  |
| 29 | a_D | | | | average duration of D periods | |  |  |
| 30 | M_D | | | | median duration of D periods | |  |  |
| 31 | s_D | | | | sum duration of all periods of D | |  |  |
| 32 | n_sgD | | | | number of periods of single D | | "Single D" refers to a waveform D that returns to C without leading directly to E1. |  |
| 33 | d_1st_E | | | | duration of the first phloem phase | |  | If there is no E, then d_1st_E = missing data |
| 34 | n_sgE1 | | | | number of periods of single E1 | |  |  |
| 35 | a_sgE1 | | | | average duration sgE1 periods | |  | If there are no sgE1 periods, then  a_sgE1 = missing data |
| 36 | m_sgE1 | | | | median duration sgE1 periods | |  | If there are no sgE1 periods, then  m_sgE1 = missing data |
| 37 | s_sgE1 | | | | sum duration of all periods of sgE1 | |  |  |
| 38 | | mx_sgE1 | | | longest period of sgE1 | |  | If there are no sgE1 periods, then  mx_sgE1 = missing data |
| 39 | | n_frE1 | | | number of periods of frE1 | |  |  |
| **NON-SEQUENTIAL VARIABLES** | | | | | | |  |  |
| Number # | | | Acronym | | Variable definition | | Notes | Value when waveform does not occur |
| 40 | | a_frE1 | | | average duration of frE1 periods | | frE1 = "E1 fraction" which is an E1 with a preceding or subsequent E2 period | If there are no frE1 periods, then  a_frE1 = missing data |
| 41 | | m_frE1 | | | median duration of frE1 periods | |  | If there are no frE1 periods, then  m_frE1 = missing data |
| 42 | | s_frE1 | | | sum duration of all periods of frE1 | |  |  |
| 43 | | mx_frE1 | | | longest period of frE1 | |  | If there are no frE1 periods, then  mx_frE1 = missing data |
| 44 | | n_E1 | | | number of periods of E1 | | E1 includes all frE1 and sgE1 |  |
| 45 | | a_E1 | | | average duration of E1 periods | |  | If there are no E1 periods, then  a_E1 = missing data |
| 46 | | m_E1 | | | median duration of E1 periods | |  | If there are no E1 periods, then  m_E1 = missing data |
| 47 | | s_E1 | | | sum duration of all periods of E1 | |  |  |
| 48 | | mx_E1 | | | longest period of E1 | |  | If there are no sE1 periods, then  mx_E1 = missing data |
| 49 | | a_1st E1_followed_E2 | | | mean duration of initial E1 in phloem phase | | only duration of initial E1 in each phloem phase; E1s that occur later in the same phloem phase are not included | If there are no E1, then  a_1st E1_followed_E2= missing data |
| 50 | | n_E12 | | | number of periods of E12 | | E12 = periods with both E1 and E2.  A single period of E12 can have a single E1 and E2 or multiple periods of E1 and E2. |  |
| 51 | | a_E12 | | | average duration of E12 periods | |  | If there are no E12 periods, then  a_E12 = missing data |
| 52 | | m_E12 | | | median duration of E12 periods | |  | If there are no E12 periods, then  m_E12 = missing data |
| 53 | | s_E12 | | | sum duration of all periods of E12 | |  |  |
| 54 | | mx_E12 | | | longest period of E12 | |  | If there are no E12 periods, then  mx_E12 = missing data |
| 55 | | n_E2 | | | number of periods of E2 | | if there are multiple periods of E2 (e.g., C-E1-E2-E1-E2-C) in the same phloem phase, each E2 counts as a separate period of E2 (e.g., in the example above, n_E2= 2) |  |
| 56 | | a_E2 | | | average duration of E2 periods | |  | If there are no E2 periods, then  a_E2 = missing data |
| 57 | | m_E2 | | | median duration of E2 periods | |  | If there are no E2 periods, then  m_E2 = missing data |
| 58 | | s_E2 | | | sum duration of all periods of E2 | |  |  |
| 59 | | a_s_E2/phloem_ph | | | mean duration of E2 per phloem phase | | s_E2/(n_sgE1+n_E12) | If there is no phloem phase (sgE1+E12) then %n_sE2/E2=missing data  If there are phloem phases but no E2 then %n_sE2/E2=0 |
| 59 | | mx_E2 | | | longest period of E2 | | Be aware: often the longest E2 is artificially terminated by end of recording and consequently is an underestimate | If there are no E2 periods, then  mx_E2 = missing data |
| 60 | | d_1st_E2 | | | duration of the 1st E2 in the recording | |  | If there are no E2 periods, then  d_1st_E2 = missing data |
| 61 | | %_E2/Tr | | | % insects with E2 | | calculated over all replicates in the treatment |  |
| 62 | | n_sE2 | | | number of periods of sE2 | | sE2 = sustained E2 = E2 > 10 min |  |
| 63 | | a_sE2 | | | average duration of sE2 periods | |  | If there are no sE2 periods, then  a_sE2 = missing data |
| 64 | | m_sE2 | | | median duration of sE2 periods | |  | If there are no sE2 periods, then  m_sE2 = missing data |
| 65 | | s_sE2 | | | sum duration of all periods of sE2 | |  |  |
| 66 | | %_sE2/Tr | | | % insects with sE2 | | calculated over all replicates in the treatment |  |

**SEQUENTIAL VARIABLES**

Sequential variables are measures of time or number of waveform periods that occur before or after a specified point in the recording. There are 3 general types of sequential variables:

1) "time to" variables: these variables calculate the time from a defined starting point to the start of a waveform. The defined starting point can be the start of the recording (variable 63), from the start of the first probe in the recording (variables 64-67, 108), or from the start of the probe to the first occurrence of a waveform in that probe (variables 109-112).

2) "time in" or "duration of" variables: These variables (variables 68-72, 110-112) calculate the total time, average time, or minimum time spent in certain activities before or after a defined waveform period. Examples: "time in C to 1stE in 1st probe with E" (variable 68), "average time in C to 1stE in probes with E" (variable 70).

3) "number of" variables: these variables (variables 73-80, 113, 115) calculate the number of certain waveforms that occur before or after a defined waveform period.

| Number # | Acronym | Variable definition | Notes | Value when waveform does not occur |
| --- | --- | --- | --- | --- |
| **"Probing”** | | | | |
| 67 | t>1Pr | time to 1st probe from start of recording |  |  |
| **“Phloem phase"** | | | | |
| 68 | t>1E | time to 1st E from start of 1st probe | E refers to phloem phase regardless of whether it has only E1 (sgE1) or has both E1 and E2 (E12) | If there is no E, then t>1E = time from start of 1st probe to end of recording |
| 69 | t>1E12 | time to the 1st E12 from start of 1st probe |  | If there is no E12, then t>1E12 = time from start of 1st probe to end of recording |
| 70 | t>1E2 | time to the 1st E2 from start of 1st probe |  | If there is no E2, then t>1E2 = time from start of 1st probe to end of recording |
| 71 | t>1sE2 | time to 1st sE2 from start of 1st probe |  | If there is no sE2, then t>1E2 = time from start of 1st probe to end of recording |
| 72 | tPr>1E/1Pr | Time from the beginning of that probe to 1st E | Total time in probe before start of 1st E in the  1st probe with E | If no there is no E, then tPr>1E/1Pr = missing data |
| 73 | tPr>1E2/1Pr | Time from the beginning of that probe to 1st E2 | Total time in probe before start of 1st E2 in the  1st probe with E | If no there is no E, then tPr>1E2/1Pr = missing data |
| 74 | tPr>1sE2/1Pr | Time from the beginning of that probe to 1st sE2 | Total time in probe before start of 1st sE2 in the  1st probe with E | If no there is no E, then tPr>1sE2/1Pr = missing data |
| 75 | tC>1E/1Pr | time in C to 1stE in 1st probe with E | Total time in C before start of 1st E in the  1st probe with E  if any F or G before 1stE, then use 2nd E | If no there is no E, then tC>1E/1Pr = missing data |
| 76 | tC>1sE2/1Pr | time in C to 1st sE2 in 1st probe with sE2 | Total time in C before start of 1st sE2 in the  1st probe with sE2  if any F or G before 1stE, then use 2nd E | If there is no sE2, then tC>1sE2/1Pr = missing data |
| 77 | atC>1E/Pr | average time in C to 1stE in probes with E | Average time in C from start of probe to 1st E in the probe. Includes only probes with E. | If there is no E, then atC>1E/Pr = missing data to 1^st^ E in the probe. includes only probes with E |
| 78 | mntC>1E/Pr | minimum time in C to 1st E in probes with E | Minimum time in C from start of probe to 1st E in the probe. Includes only probes with E | If there is no E, then mntC>1E/Pr = missing data |
| 79 | s_np.1E | total duration of nonprobing before the 1st E in the recording |  | If there is no E, then s_np.1E = total duration of nonprobing in recording |
| 80 | n_Pr>1E | number of probes before 1st E in the recording | The probe in which the 1st E occurs is included. | If there is no E, then n_Pr>1E=total number of probes in the recording |
| 81 | n_brPr>1E | number of brief probes (<3min) before 1st E in the recording | The probe in which the 1st E occurs is included (if it is brief). | If there is no E, then n_brPr>1E=total number of brief probes in the recording |
| 82 | n_Pr>1E2 | number of probes before 1st E2 in the recording | The probe in which the 1st E2 occurs is included. | If there is no E2, then n_Pr>1E2=total number of probes in the recording |
| 83 | n_Pr>1sE2 | number of probes before 1st sE2 in the recording | The probe in which the 1st sE2 occurs is included. | If there is no sE2, then n_Pr>1sE2=total number of probes in the recording |
| 84 | n_E2>1sE2 | number of E2 before 1st sE2 in the recording | if the first E2 is an sE2 (i.e., > 10 min) then n_E2>1sE2 = 0  The probe in which the first sE2 occurs is NOT included | If there are E2s but no sE2, then  n_E2>1sE2= total number of E2s.  If there are no E2s and no sE2s  then n_E2>1sE2=missing data |
| 85 | n_Pr.after1E | number of probes after 1st E in the recording | The probe in which 1st E occurs is not included | If there is no E, then n_Pr.after E1 = missing data |
| 86 | n_bPr.after1E | number of brief probes (<3min) after 1st E in the recording | The probe in which 1st E occurs is not included | If there is no E, then n_bPr.after E1 = missing data |
| 87 | n_ Pr<1sE2 | number of probes after 1st sE2 in the recording | The probe in which 1st E2 occurs is not included | If there is no sE2, then n_Pr>1sE2 = missing data |
| 88 | d_E1followedbyE2 | duration of E1 followed by E2 | all initial E1 followed by E2. | If there is no E2, then d_E1followed by E2 = missing data |

| **SEQUENTIAL VARIABLES** | |  |  |  |
| --- | --- | --- | --- | --- |
| Number # | Acronym | Variable definition | Notes | Value when waveform does not occur |
| **"Ratios and Indices"** | | | | |
| 89 | d_E1followedbysE2 | duration of E1 followed by sE2 | all initial E1 followed by sE2.  This value is an average | If there is no sE2, then d_E1 followed by sE2 = missing data |
| 90 | E2/C_ratio | E2/C ratio | (s_E2/s_C)/100 | If there are no E2 periods, then  (S_E2/s_C)/100 = 0 |
| 91 | E1_index | E1 index | (s_E1/s_E12+sgE1)*100 | If no E, then E1 index = missing data |
| 92 | frE1_ratio | E fractioning ratio | n_frE1/n_E12  frE1=allE1-sgE1 | If no E12, then n_frE1/n_E12 = missing data |
| 93 | E2_index | E2 index | Time in E2 (s_E2) / time left in the recording after start of 1st E2*100 | If no E2, then E2 index = missing data |
| 94 | %probtimeinC | % of probing time spent in C | total duration of all C (pds are included in C) / total duration of all waveforms except np |  |
| 95 | %probtimeinF | % of probing time spent in F | total duration of all F / total duration of all waveforms except np | If no F, then %probtimeinF = 0 |
| 96 | %probtimeinG | % of probing time spent in G | total duration of all G / total duration of all waveforms except np | If no G, then %probtimeinG = 0 |
| 97 | %probtimeinE1 | % of probing time spent in E1 | total duration of all E1 / total duration of all waveforms except np | If no E1, then %probtimeinE1 = 0 |
| 98 | %probtimeinE2 | % of probing time spent in E2 | total duration of all E2 / total duration of all waveforms except np | If no E2, then %probtimeinE2= 0 |
| 99 | %_sE2 | % of E2s that are sustained E2s (i.e., >10 min) | (n_sE2/ n_E12)*100 | If there are no E2s, then %n_sE2/E2=missing data  If there are E2s but no sE2 then %n_sE2/E2=0 |
| 100 | %Phloem_ph_fail | % of phloem phases that fail to achieve ingestion | (n_sgE1/(n_E12 + n_sgE1) )*100 | If there are no E1s then %n_sgE1/n_E1=missing data  If there are E1s but no sgE1 then %n_sgE1/n_E1=0 |
| **"Potential drops" (pds)**  Potential drops represent brief intracellular stylet punctures in plant cells. Recently, in some aphid species, two types of pds have been identified: "standard pds" which represent brief intracellular stylet punctures in plant cells other than sieve elements and their associated companion cells and "phloem-pds" which represent intracellular punctures of the companion cell/sieve element complex. Standard pds are associated with acquisition and inoculation of non-persistent viruses and inoculation of semipersistent viruses by aphids, and phloem pds are associated with the transmission of some phloem-limited viruses by aphids. If two types of pds have been identified in the study system, then the researcher has the option of separately marking the two types of pds: standard pds (pd, variable code 8) and phloem-pds (P-pd, variable code 11), and they will be analyzed separately as indicated in the variable list below. If a study is on a system where two types of pds have not been identified (or if the researcher chooses not to separately mark standard pds and phloem pds), then all pds should be marked as pds (pd, variable code 8) and the calculated pd variables refer to all pds regardless of what cell type is punctured. | | | | |
| **“Standard potential drops (pd)”** | | | | |
| **NON-SEQUENTIAL VARIABLES** | | | | |
| Number # | Acronym | Variable definition | Notes | Value when waveform does not occur |
| 101 | n_pd | number of pds |  |  |
| 102 | n_pd/minC | number of pds per minute of pathway phase | = n_pd/s_C  This variable is more instructive than n_pd | if there is no pd, then n_pd/minC = 0 |
| 103 | a_pd | average duration of pds |  | if there is no pd, then a_pd = missing data |
| 104 | m_pd | median duration of pds |  | if there is no pd, then m_pd = missing data |
| 105 | s_pd | sum duration of all pds |  |  |
| **“Pd sub-phases”**  Pd subphases II-1, II-2 and II-3 are characteristic of aphid pds; this section applies only to aphids, but caution: subphases of potential drops are often difficult to analyze due to difficulty precisely identifying the transition of one subphase to the next. The total durations of subphases are brief, so error introduced by difficulty precisely identifying the start and end can have a large impact on its estimated duration. The transition of subphase II-1 to II-2 is often especially difficult to precisely identify. This difficulty is compounded by subphase II-1 possibly be composed of 2 subphases. Consequently these variables might not be reliable. Transition of subphase II-2 to II-3 is often much clearer, which can result in a more accurate measurement of the duration of II-3. If the user chooses mark only pds and not pd subphases, then variables 99-107 will appear as missing data. | | | | |
| 106 | a_pd II-1 | average pd II-1 duration |  | if there is no pd, then a_pd II-1 = missing data |
| 107 | m_pd II-1 | median pd II-1 duration |  | if there is no pd, then m_pd II-1 = missing data |
| 108 | s_pd II-1 | sum of time pd II-1 periods |  |  |
| 109 | a_pd II-2 | average pd II-2 duration |  | if there is no pd, then a_pd II-2 = missing data |
| 110 | m_pd II-2 | median pd II-2 duration |  | if there is no pd, then m_pd II-2 = missing data |
| 111 | s_pd II-2 | sum of time pd II-2 periods |  |  |
| 112 | a_pd II-3 | average pd II-3 duration |  | if there is no pd, then a_pd II-3 = missing data |
| 113 | m_pd II-3 | median pd II-3 duration |  | if there is no pd, then m_pd II-3 = missing data |
| 114 | s_pd II-3 | sum of time pd II-3 periods |  |  |
| **SEQUENTIAL VARIABLES** | | | | |
| Number # | Acronym | Variable definition | Notes | Value when waveform does not occur |
| 115 | t>1pd | time to 1st pd | time from start of 1st probe to 1st pd in the recording | if there is no pd, t>1pd = time from start of 1st probe to end of recording |
| 116 | t>1pd/1Pr | time to 1st pd in 1st probe | time to 1st pd from start of probe in which the 1st pd occurs | if there is no pd, then t>1pd/1Pr = missing data |
| 117 | at>1pd/Pr | average time to 1st pd in a probe for all probes with pds | time to 1st pd is from start of the probe in which it occurs | if there is no pd, then at>1pd/Pr = missing data |
| 118 | m_Pr>1pd | median time to 1st pd in a probe for all probes with pds | time to 1st pd is from start of the probe in which it occurs | if there is no pd, then m_Pr>1pd = missing data |
| 119 | mnt_1pd/1pd | Minimum time to 1st pd in a probe among all probes with pds | time to 1st pd is from start of the probe in which it occurs | if there is no pd, then mnt_1pd/1pd = missing data |
| 120 | n_pd/1Pr | number of pds in 1st probe |  |  |
| 121 | %_Pr_pd | % probes with at least one pd |  | if there is no pd, then %_Pr_pd = 0 |
| 122 | n_Pr>1pd | number of probes before 1st pd |  | If there is no pd then n_Pr>1pd= number of probes in the recording |
| 123 | s_pdII-3/5pd | sum duration of II-3 in 1st 5 pds |  | If there are no pds then  s_pdII-3/5pd=missing data |
|  | | | | |
| **"Phloem-pds":** represent brief intracellular stylet punctures of the companion cell/sieve element complex | | | | |
| Number # | Acronym | Variable definition | Notes | Value when waveform does not occur |
| 124=28 | n_p-pd | number of phloem pds |  |  |
| 125=29 | a_p-pd | average duration of p-pd |  |  |
| 126=30 | m_p-pd | median duration of p-pd |  |  |
| 127=31 | s_p-pd | Total duration summed over all phloem pds |  |  |
|  | | | | |
