# Supplementary material for: Novel program for automatic calculation of EPG variables
Source: J Insect Sci. 2024 Jun 28;24(3):28. doi: 10.1093/jisesa/ieae063 (PMC11212364; doi:10.1093/jisesa/ieae063)
Supplement: ieae063_suppl_Supplementary_Table_S2 [file ieae063_suppl_supplementary_table_s2.docx]

**Table S2. List of mathematical formulas used to calculate the EPG variables**

The notation used in the mathematical definitions is explained as follows:

$w_{\left( i, j|k,p \right)}$: is the wave *w* that occupies the position *j* within the recording *i* and can also be identified in reference to the probe *k* belonging to the recording *i* as the wave *w* that occupies the position *p*

$w_{\left( i, j \right)}$*:* in some cases, it will be necessary to express the wave *w* according to its position *j* in the recording *i*

${w'}_{\left( k,q \right)}$*:* in other cases, it will be necessary to express the wave *w* according to its position *q* in the probe *k* and in this case, it will be identified as *w’*

w: refers to a wave within a recording

w’: refers to a wave within a probe

i: insect (i → N)

j: refers to a wave in the sequence of the recording (j → n)

k: refers to a probe within a recording (k → P)

q: the wave in the sequence of a probe (q → p)

**NON- SEQUENTIAL VARIABLES**

| Variable definition | Formulas |
| --- | --- |
| number of periods of the waveform  (where *w_i_* represents the occurrence of wave *w* during the i-th period) | $n_{w}=\sum_{j=1}^{n} w_{(j\to w)}$ |
| average duration of the waveform  (where *d_wi_* is the duration of the wave *w* in the i-th period) | $a_{w}=\frac{\sum_{j=1}^{n} d_{wj}}{n_{w}}$ |
| median duration of the waveform | ${med}_{w}=med\{\left( d_{\left( i,w1 \right)} \right),\left( d_{\left( i,w2 \right)} \right),\ldots,\left( d_{\left( i,wn \right)} \right)\}$ |
| sum duration of all periods of the waveform | $s_{w}=\sum_{j=1}^{n} d_{wj}$ |
| maximum waveform period duration | ${mx}_{w}=\max_{j=1}^{n}\left( d_{wj} \right)$ |
| duration of the first phloem phase | $d_{1E}=t_{end(1E)}-t_{start(1E)}$ |
| mean duration of initial E1 in phloem phase | $\bar{d}_{1E1E2}=\sum_{j=1}^{n} \frac{d_{1E1(j\to E1E2)}}{n_{(j\to1E1E2)}}$ |
| duration of the 1st E2 in the recording | $d_{1E2}{=t}_{end,1E2}-t_{start,1E2}$ |
| % insects with E2 or sE2/Tr | $p_{E2}=\frac{n_{E2}}{N}\cdot100$ or $p_{sE2}=\frac{n_{sE2}}{N}\cdot100$ |

**SEQUENTIAL VARIABLES**

| Variable definition | Formulas |
| --- | --- |
| time to 1st probe from start of recording | $t_{start\to1PR}=d_{1NP}$ |
| time to 1st E from start of 1st probe | $t_{E'(1,E)}=\sum_{q=1}^{E} t_{w'(1,q)}$ |
| time to the 1st E12 from start of 1st probe | $t_{E12'(1,E12)}=\sum_{q=1}^{E12} t_{w'(1,q)}$ |
| time to the 1st E2 from start of 1st probe | $t_{1E2'(1,E2)}=\sum_{q=1}^{E2} t_{w'(1,q)}$ |
| time to 1st sE2 from start of 1st probe | $t_{1sE2'(1,sE2)}=\sum_{q=1}^{sE2} t_{w^{'}}(1,q)$ |
| Time from the beginning of that probe to 1st E | $t_{1E'(k\to E)}=\sum_{q=1}^{E} t_{w'(k\to E,q)}$ |
| Time from the beginning of that probe to 1st E2 | $t_{1E2'(k\to E2)}=\sum_{q=1}^{E2} t_{w'(k\to E2,q)}$ |
| Time from the beginning of that probe to 1st sE2 | $t_{1sE2'(k\to sE2)}=\sum_{q=1}^{sE2} t_{w'(k\to sE2,q)}$ |
| time in C to 1stE in 1st probe with E | $t_{w'(1pr,E\neg(F^G))}=\sum_{q=C}^{E} t_{q}$ |
| time in C to 1st sE2 in 1st probe with sE2 | $t_{w'(1pr,sE2\neg(F^G))}=\sum_{q=C}^{sE2} t_{q}$ |
| average time in C to 1stE in probes with E | $\bar{t}_{C(k,1E)}=\frac{\sum_{q=C}^{1E} t_{c(k\to E,q)}}{\sum_{k}^{P} n_{(k\to E)}}$ |
| minimum time in C to 1st E in probes with E | $t_{min(k\to E,E)}={{min}_{k=1}^{P}t}_{c\left( k\to E,1E \right)}$ |
| total duration of non-probing before the 1st E in the recording | $t_{Np\left( i,j \right)}=\sum_{j=1E}^{1} {Np}_{(i,j)}$ |
| number of probes before 1st E in the recording | $n_{\Pr\left( i,1E \right)}=\sum_{j=1E}^{1} n_{(i,j\to Pr)}$ |
| number of brief probes (<3min) before 1st E in the recording | $n_{Pr\to t<3 \left( i,\leftarrow1E \right)}=\sum_{j=1E}^{1} n_{t<3(i,j\to Pr)}$ |
| number of probes before 1st E2 in the recording | $n_{Pr\to t<3 \left( i,\leftarrow1E \right)}=\sum_{j=1E}^{1} n_{t<3(i,j\to Pr)}$ |
| number of probes before 1st sE2 in the recording | $n_{\Pr\left( i,\leftarrow sE2 \right)}=\sum_{j=1sE2}^{1} n_{(i,j\to Pr)}$ |
| number of E2 before 1st sE2 in the recording | $n_{E2 \left( i,\leftarrow sE2 \right)}=\sum_{j=1sE2}^{1} n_{(i,j\to E2)}$ |
| number of probes after 1st E in the recording | $n_{\Pr\left( i,E\to\right)}=\sum_{j=1}^{1E} n_{(i,j\to Pr)}$ |
| number of brief probes (<3min) after 1st E in the recording | $n_{Pr\to t<3 \left( i,1E\to\right)}=\sum_{j=1}^{1E} n_{t<3(i,j\to Pr)}$ |
| number of probes after 1st sE2 in the recording | $n_{(i,1sE2\to)}=\sum_{j=1}^{1sE2} n_{\left( i,j\to Pr \right)}$ |
| duration of E1 followed by E2 | $d_{(i,j\to E1-E2)}=\sum_{j=1}^{n} t_{E1(i,j\to E1-E2)}$ |
| duration of E1 followed by sE2 | $d_{(i,j\to E1-sE2)}=\sum_{j=1}^{n} t_{E1(i,j\to E1-sE2)}$ |
| E2/C ratio | $\frac{t_{E2}}{t_{C}}=\frac{\sum_{j=1}^{n} t_{E2(i,j\to E2)}}{\sum_{j=1}^{n} t_{C(i,j\to C)}}\cdot100$ |
| E1 index | ${E1}_{index}=\frac{\sum_{j=1}^{n} d_{(j\to E1)}}{\sum_{j=1}^{n} {[d}_{(j\to(E12)}+d_{(j\to(E1,\neg\left( E1E2^E2E1 \right)))}]}\cdot100$ |
| E fraction ratio | $E_{fr}=\frac{\sum_{j=1}^{n} {[n}_{E1(j\to E1)]}-n_{E1(j\to(E1,\neg(E1E2^E2E1)))}}{\sum_{j=1}^{n} n_{E12(j\to E12)}}$ |
| E2 index | ${E2}_{index}=\frac{\sum_{j=1}^{n} d_{(j\to E2)}}{d_{j=n}-\sum_{j=1}^{1E2} t_{wj}}\cdot100$ |
| % of probing time spent in waveform (C, F,G,E1, E2) | $P{robing}_{C\%}=\frac{\sum_{j=1}^{n} d_{(j\to C)}}{\sum_{j=1}^{n} d_{(j\to\left( \neg Np \right))}}\cdot100$  $P{robing}_{F\%}=\frac{\sum_{j=1}^{n} d_{(j\to F)}}{\sum_{j=1}^{n} d_{(j\to\left( \neg Np \right))}}\cdot100$  $P{robing}_{G\%}=\frac{\sum_{j=1}^{n} d_{\left( j\to G \right)}}{\sum_{j=1}^{n} d_{\left( j\to\left( \neg Np \right) \right)}}\cdot100$  $P{robing}_{E1\%}=\frac{\sum_{j=1}^{n} d_{(j\to E1)}}{\sum_{j=1}^{n} d_{(j\to\left( \neg Np \right))}}\cdot100$  $P{robingE2}_{\%}=\frac{\sum_{j=1}^{n} d_{(j\to E2)}}{\sum_{j=1}^{n} d_{(j\to\left( \neg Np \right))}}\cdot100$ |
| % of E2s that are sustained E2s (i.e., >10 min) | ${E2}_{\%}=\frac{\sum_{j=1}^{n} n_{w(j\to sE2)}}{\sum_{j=1}^{n} n_{w(j\to E2)}}\cdot100$ |
| % of phloem phases that fail to achieve ingestion | ${ph}_{\%}=\frac{\sum_{j=1}^{n} n_{w(j\to\left( E1,\neg\left( E1E2^E2E1 \right)) \right)}}{\sum_{j=1}^{n} {[n}_{w(j\to(E12)}+n_{s(j\to(E1,\neg\left( E1E2^E2E1 \right)))}]}\cdot100$ |
| number of pds per minute of pathway phase | $\dot{n}_{pd}=\frac{\sum_{j=1}^{n} n_{w(j\to pd)}}{\sum_{j=1}^{n} d_{(j\to C)}}$ |
| time to 1st pd | $t_{1pd}=\sum_{j=1}^{1pd} t_{j}$ |
| time to 1st pd in 1st probe | $t_{(1pd\to k=1)}=\sum_{j=1}^{1pd} t_{j}$ |
| average time to 1st pd in a probe for all probes with pds | $\bar{t}_{1pd}=\frac{\sum_{k=1}^{P} \sum_{q=1}^{1pd} t_{(k\to pd,q)}}{n_{k\to pd}}$ |
| median time to 1st pd in a probe for all probes with pds | ${med}_{t(k,1pd)}=med\{t\left( k_{1,1pd} \right),t\left( k_{2,1pd} \right),\ldots,t\left( k_{P,1pd} \right)\}$ |
| Minimum time to 1st pd in a probe among all probes with pds | $t_{min,1pd (k\to pd)}=min\{t\left( k_{1,1pd} \right),t\left( k_{2,1pd} \right),\ldots,t\left( k_{P,1pd} \right)\}$ |
| number of pds in 1st probe | $n_{pd(k=1)}=\sum_{q}^{p} n_{pd(k=1,q)}$ |
| % probes with at least one pd | ${Pr}_{\%pd}=\frac{\sum_{k=1}^{P} \sum_{q=1}^{p} n_{pd(k\to pd,q)}}{\sum_{k=1}^{P} n_{k(k,q\to Pr)}}\cdot100$ |
| number of probes before 1st pd | $n_{\Pr\left( \leftarrow1pd \right)}=\sum_{1pd}^{k=1} n_{pr(k)}$ |
| sum duration of II-3 in 1st 5 pds | $d_{II-3}=\sum_{j=1}^{n=5pd} t_{(j\to pd(II-3))}$ |
